# Pathological Assessment of Rectal Cancer after Neoadjuvant Chemoradiotherapy: Distribution of Residual Cancer Cells and Accuracy of Biopsy

**DOI:** 10.1038/srep34923

**Published:** 2016-10-10

**Authors:** Lin Xiao, Xin Yu, Wenjing Deng, Huixia Feng, Hui Chang, Weiwei Xiao, Huizhong Zhang, Shaoyan Xi, Mengzhong Liu, Yujia Zhu, Yuanhong Gao

**Affiliations:** 1 Department of Oncology, Section II, Jiangmen Central Hospital, Affiliated Jiangmen Hospital of Sun Yat-sen University, Jiangmen, 529030,China; 2 Department of Radiation Oncology, Sun Yat-sen University Cancer Center, China; 3 State Key Laboratory of Oncology in South China, Collaborative Innovation Center of Cancer Medicine, Guangzhou, 510060, China; 4 Department of Oncology, Section III, Jiangmen Central Hospital, Affiliated Jiangmen Hospital of Sun Yat-sen University, Jiangmen, 529030,China; 5 Department of Pathology, Sun Yat-sen University Cancer Center, Guangzhou, China

## Abstract

We investigated the distribution of residual cancer cells (RCCs) within different layers of the bowel wall in surgical specimens and the value of biopsies of primary rectal lesion after preoperative volumetric modulated arc therapy (VMAT) with concurrent chemotherapy in patients with rectal cancer. Between April 2011 and April 2013, 178 patients with rectal cancer who received preoperative VMAT, concurrent chemotherapy, and surgery were evaluated; 79 of the patients received a biopsy of the primary lesion after chemoradiotherapy and prior to surgery. The distribution of RCCs in the surgical specimens and the sensitivity and specificity of the biopsy of primary rectal lesions for pathological response were evaluated. Fifty-two patients had a complete pathological response in the bowel wall. Of the 120 patients with ypT2-4, the rate of detection of RCCs in the mucosa, submucosa, and muscularis propria was 20%, 36.7%, 69.2%, respectively. The sensitivity and specificity of biopsies of primary rectal lesions was 12.9% and 94.1%, respectively. After chemoradiotherapy, the RCCs were primarily located in the deeper layers of the bowel wall, and the biopsy results for primary rectal lesions were unreliable due to poor sensitivity.

Preoperative 3- or 4-field radiation with concurrent fluoropyrimidine-based chemotherapy (5-FU or capecitabine) and subsequent total mesorectal excision (TME) is currently the standard treatment for locally advanced rectal cancer (LARC, i.e. T3/T4 or N positive)^1^,^2^. However, the tumor response to neoadjuvant chemoradio -therapy (neoCRT) varies significantly among individuals: 15–27% of the patients achieve a pathological complete response (pCR), a partial response is seen in 54–75% of patients and others show no response at all^3^,^4^. Increasing clinical evidence supports the idea that patients who achieve a pCR after neoCRT exhibit better local control, disease-free survival and overall survival than those who do not achieve a pCR^3^,^5^. Some studies have shown promising results with “watch and wait” approaches in carefully selected patients who developed a clinical complete response (cCR) after neoCRT^6^,^7^,^8^. This approach of organ-preservation helps patients avoid the risks of surgical morbidity and mortality, such as the occurrence of anastomotic fistula, intestinal obstruction, and sexual and urinary dysfunction^9^,^10^. However, a key determinant that affects how this nonsurgical treatment approach can be implemented safely is the accurate identification of patients with a cCR and a guarantee of the consistency of cCR and pCR. In the study by Bujko K, 56% of the patients with cCR after neoCRT had residual cancer cells (RCCs) in their bowel walls^11^. Approximately 6.5% to 8.3% of patients who developed non-cCR after neoCRT developed pCR^8^,^12^. Currently, neither diffusion-weighted magnetic resonance imaging (MRI) nor ^18^F-fluorodeoxy-glucose positron emission tomography/computed tomography (^18^F- FDG PET/CT) can accurately identify patients with pCR^4^,^13^,^14^. A histopathological assessment of a surgical specimen remains the only reliable method for the diagnosis of patients with pCR after neoCRT^15^. A biopsy of the primary rectal lesion after neoCRT, which is one of the easiest and most straight-forward approaches for the assessment of response to neoCRT^16^, is used to judge whether a patient has achieved a cCR. This process requires no visible RCCs in the biopsy specimen^6^,^8^,^12^,^17^, but the reliability of biopsy is still unsatisfactory^16^,^18^,^19^.

An understanding of the distribution of RCCs within the different layers of the bowel wall (mucosa, submucosa, muscularis propria, and subserosa) of surgical specimens after neoCRT may potentially improve the value of biopsy of the primary rectal lesion (such as guiding clinicians in deciding how and where to perform the biopsy). An understanding of RCCs may also improve the consistency of cCR and pCR after neoCRT in patients with mid to low-lying rectal cancer who wish to be treated with a non-surgical approach. Additionally, this understanding may also facilitate the exploration of the underlying mechanisms of resistance to chemoradiotherapy. Eventually, all of this information will aid in the development of better strategies for the preoperative identification of patients with potential pCR and for the implementation of individualized treatments. However, related reports in this field are rare.

Duldulao MP *et al.*^20^ reported that in rectal cancer specimens of patients treated with preoperative conventional radiation plus concurrent 5-FU-based chemotherapy, RCCs were distributed preferentially in the muscularis propria and subserosa layers. It is not clear whether different radiotherapy techniques, such as intensity-modulated radiation therapy (IMRT) and volumetric modulated arc therapy (VMAT), could significantly affect the distribution of RCCs in the bowel wall after neoCRT. However, several studies have shown that IMRT and VMAT may be used with neoCRT for the treatment of patients with LARC, and that these therapeutic modalities lead to high pCR rates with an acceptable toxicity profile^21^,^22^.

In this study, we investigated the distribution of RCCs and the value of biopsy through an assessment of sensitivity and specificity after preoperative VMAT plus concurrent chemotherapy. We examined the distribution of RCCs in different layers of the bowel wall in 178 patients with rectal cancer who were treated with this mode of neoCRT. For 79 patients, we also compared the pathology results of the biopsy specimens of primary rectal lesions after chemoradiotherapy and prior to surgery with those of their corresponding surgical specimens.

## Materials and Methods

### Ethical approval and Informed consent

All procedures performed in the study were in accordance with the ethical standards of Sun Yat-sen University Cancer Center’s Ethics Committee and with the 1964 Helsinki declaration and its later amendments or comparable ethical standards. All the study protocols were approved by the Sun Yat-sen University Cancer Center’s Ethics Committee. Informed consent for data handling for research purposes at the time of the patient’s first visit was obtained from all individual participants included in the study.

#### Patients

All consecutive patients with rectal carcinoma who received preoperative VMAT with concurrent chemotherapy and subsequent surgery from April 2011 to April 2013 at the Sun Yat-sen University Cancer Center were systematically studied. The pretreatment clinical staging tools included endorectal ultrasonography (EUS), pelvic MRI and/or computed tomography (CT) scans, chest CT and/or X-ray, abdominal CT and/or MRI and/or ultrasound. In cases of staging discrepancy, the higher stage was used. The 7th edition of the TNM staging standard of the American Joint Committee on Cancer (AJCC) was used. The distance from the inferior pole of the tumor to the anal verge was measured.

### Treatment

#### Radiotherapy

All radiotherapy was administered via VMAT, which is a dynamic form of IMRT and has a similar dosage distribution and greatly reduced mean treatment time compared with classical static IMRT^23^. Patients with a full bladder underwent CT-based simulation using 3-mm slices. The gross tumor volume (GTV), which was defined as the primary tumor and involved lymph nodes as observed by pelvic MRI/CT, and the clinical target volume (CTV), which was defined according to the principles recommended by Myerson RJ^24^, were contoured on axial CT scan slices. Regarding the planning target volume (PTV), PTV1 was obtained by adding 6- to 9-mm uniform margins to the GTV, whereas the PTV2 was obtained by adding 6- to 9-mm uniform margins to the CTV. The prescribed radiation doses were 50 Gy to PTV1 and 46 Gy to PTV2. The radiation was delivered in 25 daily fractions (5 days per week) of 2.0 and 1.84 Gy, respectively. The beam energy was 6~8 MV. All VMAT plans were set up in a single arc modality, which was generated with Manaco 2.0 TPS (Elekta Corporation, Sweden), and delivered with an Elekta linear accelerator (linac). The 100% isodose line was planned to encompass more than 98% of the PTV as a planning goal. None of the PTV was allowed to receive more than 110% of the prescribed dose.

#### Chemotherapy

The primary chemotherapy protocol consisted of CapeOX (capecitabine plus oxaliplatin). During radiotherapy, CapeOX consisted of oral capecitabine (1,000 mg/m^2^) administered twice daily on days 1–14 plus a 2-hour intravenous infusion of oxaliplatin (100 mg/m^2^) on day 1 (every 3 weeks); during neoadjuvant and adjuvant chemotherapy, the dose of oxaliplatin was increased to 130 mg/m^2^, whereas the dose of capecitabine was unchanged. Other protocols included single-agent capecitabine, mFOLFOX6 (5-FU with oxaliplatin and leucovorin), and FOLFIRI (5-FU with irinotecan and leucovorin).

#### Biopsy, Surgery and Pathology

All patients underwent a reassessment of the tumor clinical stage 4 to 5 weeks after chemoradiotherapy and prior to surgery with EUS/colonoscopy and pelvic MRI/CT. A biopsy was performed simultaneously. When an endoscopic biopsy was performed, neither anesthesia nor sedation was provided unless the patient experienced intolerable pain. Specimens were obtained at the location of the primary lesion, the residuals of which were visible after chemoradiotherapy. Presurgical and postchemo- radiotherapy images from pelvic MRI or CT scans were not used as a reference for the biopsy. Four to seven tissue samples from different locations within the residual primary lesion were obtained from each patient. No staining was performed before sampling. Surgery based on the TME principle was performed 6–8 weeks after the completion of radiotherapy. The extent of the residual tumor in the resected specimens was classified according to the 7th edition of the International Union Against Cancer TNM staging system. For all patients, histological sections were generated from all biopsy specimens obtained after neoCRT and from surgical specimen blocks, which were processed, prepared, stained with hematoxylin and eosin, and examined independently by 2 pathologists. A pCR was defined as the absence of any remaining viable cancer cells (not including acellular mucin pools) in all surgical resection specimens (including the primary rectal lesion and regional lymph nodes) after neoCRT (i.e., ypT0N0M0). An R0 resection was defined as a complete tumor resection with all negative margins. An R1 resection was defined as the presence of negative gross margins with positive microscopic margins.

#### Statistical analysis

Statistical analyses were performed using SPSS 16.0 statistical software (SPSS, Inc., Chicago, IL, USA). Between-group proportional differences were compared using Fisher’s exact test. A two-sided *P-*value ≤ 0.05 was considered statistically significant. The sensitivity of biopsy for the prediction of the pathological response was calculated according to the numbers of positive RCCs detected with biopsies/numbers of positive RCCs detected after surgery; the specificity was determined from the numbers of RCC-biopsy negative cases/numbers of RCC- negative cases detected after surgery.

## Results

In this study, 204 consecutive patients with rectal carcinoma were treated with preoperative VMAT and concurrent chemotherapy from April 2011 to April 2013 at Sun Yat-sen University Cancer Center. Twenty-six patients who did not undergo surgery for various reasons (heart or lung complications, 5 cases; inoperable disease, 3 cases; financial constraints, 3 cases; refusal of colostomy, 6 cases; others, 9 cases) after neoCRT were excluded. The remaining 16 patients with distant metastases at diagnosis who received palliative surgery and 162 patients with LARC (22 IIa cases, 11 IIb cases, 5 IIc cases, 1 IIIa case, 58 IIIb cases, 65 IIIc cases) who underwent radical surgery were systematically analyzed. In total, 124 of the patients were men and 54 were women; the median age was 56 years (range, 23–84 years). Regarding the primary tumor stage, 1 case had T1 disease, 2 cases had T2 disease, 73 cases had T3 disease, and 102 cases had T4 disease; in regards to the clinical lymph node staging, 40 cases had N0 disease, 46 cases had N1 disease, and 92 cases had N2 disease.

In all, 90 cases presented with low-lying rectal cancer (distance to anal verge ≤5 cm), 74 cases presented with middle rectal cancer (5.0 cm < distance to anal verge ≤ 10 cm), and 14 cases presented with high rectal cancer (distance to anal verge >10 cm).

A total of 110 patients received 1 to 2 cycles of CapeOX or capecitabine, whereas 8 patients received 2 to 6 cycles of mFOLFOX6/FOLFIRI chemotherapy prior to radiotherapy. All patients completed the concurrent chemoradiotherapy regimen (161 received 1 to 2 cycles of CapeOX, and 17 received 2 to 3 cycles of non-CapeOX protocols). The median duration of chemoradiotherapy was 36 days (range, 31–57 days). Eighty-one patients received an additional preoperative cycle of chemotherapy after the completion of radiotherapy.

Surgery was performed at a median interval of 53 days after chemoradiotherapy (range, 24–86 days). The surgical procedures included low anterior resection (110 cases), abdominoperineal resection (51 cases), Hartmann’s procedure (8 cases), coloanal anastomosis (3 cases), and other procedures (6 cases). The rate of R0 resection was 98.3% (175/178), and 3 patients underwent R1 resection. The pathological stages of the patients were as follows: 52 cases with ypT0 disease (of these 1 had pN1a disease and 1 had uncertain lymph node staging, i.e., pNx), 2 cases with ypTis disease, 4 cases with ypT1 disease, 38 cases with ypT2 disease, 77 cases with ypT3 disease, and 5 cases with ypT4 disease; 140 cases with ypN0 disease, 30 cases with ypN1 disease, 6 cases with ypN2 disease, and 2 cases with ypNx disease; 50 cases (28.1%) with ypT0N0M0 disease (i.e., ypCR), 2 cases with ypTisN0M0, 36 cases with ypI, 46 cases with ypII (43 IIa cases, 1 IIb case, 2 IIc cases), 30 cases with ypIII (5 IIIa cases, 21 IIIb case, 4 IIIc cases), 13 cases with ypIV (11 IVa cases, 2 IVb cases), and 1 case with ypT0NxM0. The total ypCR rate for the 162 patients with LARC was 30.2% (49/162).

**Figure 1:**
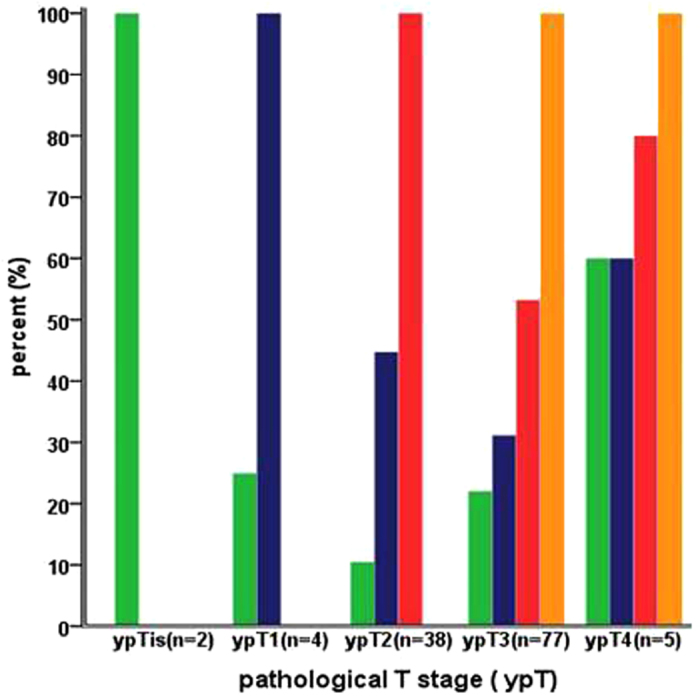
Distribution of residual cancer cells in the different layers of the bowel wall according to different ypT stage.

The overall distribution of RCCs in each layer of the bowel wall for the 178 patients was as follows: 27 (15.2%) had RCCs in the mucosa, 48 (27%) had RCCs in the submucosa, 83 (46.6%) had RCCs in the muscularis propria, and 82 (46.1%) had RCCs in the subserosa/perirectal fat. The distribution of RCCs in different layers of the bowel wall in the 126 patients with ypTis-4 tumors is shown in Fig. 1. Of the 120 patients with ypT2-4 tumors, 24 (20%) had RCCs in the mucosa, 44 (36.7%) had RCCs in the submucosa, 83 (69.2%) had RCCs in the muscularis propria.

**Figure 2:**
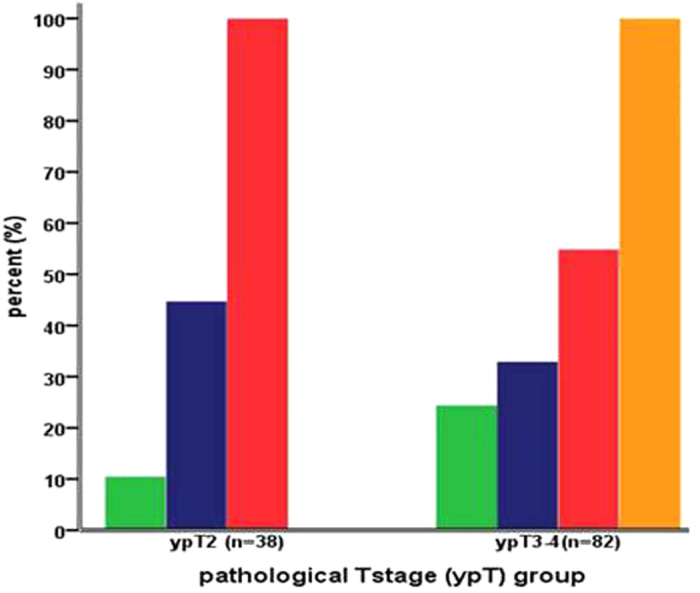
Comparison of distribution of residual cancer cells in different layers of the bowel wall between ypT2 and ypT3-4 tumor.

No significant difference was observed in the rate of detection of RCCs in the mucosa (10.5% vs. 24.4%, *P* = 0.09) and submucosa (44.7% vs. 32.9%, *P* = 0.228) of the bowel wall between patients with ypT2 and ypT3-4 tumors. Although the rate of detection of RCCs in the muscularis propria of the bowel wall in patients with ypT2 tumors was significantly higher than that in patients with ypT3-4 tumors (100% vs. 54.9%, *P* < 0.001), as shown in Fig. 2.

Seventy-nine patients (73 with LARC, 6 with stage IV disease) received an EUS exam and a simultaneous biopsy of the primary rectal lesion at a median interval of 37 days (range, 24–69 days) after the completion of neoCRT. Table 1 shows the comparison of the corresponding pathological results between the biopsy specimens and the surgical specimens.

## Discussion

Our results showed that RCCs in the rectal cancer specimens of patients treated with preoperative VMAT and concurrent chemotherapy were also primarily distributed within or below the muscularis propria; the sensitivity of biopsy for the detection of RCCs in the primary rectal lesions after neoCRT was very low. These findings will be valuable in guiding clinical practice.

Most RCCs were distributed in the layer of the muscularis propria and subserosa/perirectal fat in the bowel wall after chemoradiotherapy, which was similar to what was reported by Duldulao MP *et al.*^20^, whose neoCRT strategy consisted of preoperative conventional radiation with concurrent 5-FU-based chemotherapy. Of the 120 patients with ypT2-4 tumors, the rate of detection of RCCs in the mucosa (20%) was slightly higher than that reported by Duldulao MP *et al.*^20^ (13%), but the rates of detection of RCCs in the submucosa (36.7%) and muscularis propria (69.2%) were lower than those reported by Duldulao MP *et al.* (submucosa, 56%; muscularis propria, 98%)^20^. By contrast, the proportion of patients with cT4 (57.3%) in our study was significantly higher than that reported by Duldulao MP *et al.* (3%)^20^. Theoretically, these differences may be related to the dosimetric advantage of the VMAT technique. The 98% isodose line in every treatment plan could encompass more than 98% of the PTV, which is impossible to obtain in conventional 2-dimensional treatment plans; however, it should be noted that the dosimetry advantage of VMAT did not significantly affect the overall distribution tendency of RCCs in the bowel. Different chemotherapy regimens and the interval between the completion of chemoradiotherapy and surgery may also have contributed to these differences^25^,^26^. No significant difference was observed in the distribution of RCCs in the mucosa and submucosa of the bowel wall between ypT2 and ypT3-4 tumors. This result further suggested that the RCCs were preferentially distributed in the deeper layers of the bowel wall (muscularis propria and subserosa) and not on the surface of the bowel wall (mucosa and submucosa). This unequal distribution of RCCs in different layers of the bowel wall may be attributed to many factors. For example, the pretreatment classification of T stage and the intrinsic distribution differences in the pretreated cancer cells in different layers of the bowel wall may affect the distribution of RCCs. Duldulao MP *et al.*^20^ found that patients with cT2 tumors were more likely to have a pCR than patients with cT3 or cT4 tumors. The differences in dose intensity in the amount of chemoradiotherapy received by the cancer cells in the bowel wall may also be a factor. Furthermore, the intrinsic heterogeneity in cancer cells, which is generated by genetic alterations, the differences in the tumor microenvironment, and the existence of cancer stem cells may be the most important factors that affect the response to chemoradiotherapy and the distribution of RCCs^20^,^27^. The presence and distribution of RCCs may be the main underlying mechanism of resistance to chemoradiotherapy and may not be significantly affected by different chemoradiotherapeutic modalities.

One of the most interesting findings of this study was that the pathological results of biopsy specimens after neoCRT were usually unreliable due to the poor sensitivity of biopsy (12.9%) and the low concordance rate (30.4%) between biopsy specimens and surgical specimens; nevertheless, the specificity of biopsy was 94.1%. The biopsy of primary rectal lesions after neoCRT could not be used to accurately predict the existence of cancer cells. The rate of detection of RCCs in the mucosa and submucosa, which were the main areas sampled by forceps biopsy after neoCRT, was only 15.2% and 27%, respectively. However, most RCCs were located in deeper layers of the bowel wall, and thus it would be difficult to sample these areas by conventional biopsy. These findings may help to clarify why the sensitivity of biopsy was so low. In our study, only those patients with visible residual tumors in the primary rectal lesion after neoCRT underwent a biopsy, in spite of the residual extent after tumor downsizing. This reason may be partly why the sensitivity of the biopsy was lower than that reported by Perez RO *et al.*^16^, whose sensitivity result of postchemoradiotherapy biopsy of primary rectal lesions in 39 patients with significant tumor downsizing, but who did not achieve a cCR, was 69%. Another reason may be related to the interval between the biopsy and the end of neoCRT. The median interval was 37 days (range, 24–68 days) in our study, whereas all patients underwent a biopsy assessment 8 weeks after neoCRT in the study by Perez RO *et al.*^16^. In one retrospective review of 14 unselected patients who underwent postchemoradiotherapy biopsy, the negative predictive value of biopsy was as low as 36%^19^. Another study that prospectively evaluated postchemoradiotherapy endoscopic biopsies of primary rectal lesions to predict the pCR of rectal cancer patients was discontinued for its disappointing preliminary results. In that study, the overall concordance rate between postchemoradiotherapy biopsies and surgical specimens was 50% and none of postchemoradiotherapy biopsies showed any evidence of cancer in the first 22 patients^28^. Although the reliability of these results from the aforementioned studies may be limited due to very small sample sizes, they were all in agreement with our results in that biopsy of primary rectal lesions after neoCRT was unreliable to some extent. It is often very difficult for a conventional endoscopic biopsy to obtain a full-thickness sample that would provide adequate information of RCCs in the bowel wall of the primary rectal lesion after neoCRT. However, even with the use of a novel, minimally invasive method of incisional biopsy as a tool to restage rectal cancers after neoCRT, the sensitivity of incisional biopsy in the detection of RCCs was only 50%; obviously, incisional biopsy is not appropriate as a stand-alone method to determine whether patients are suited for the “watch and wait” technique^29^. Moreover, clinically undetectable residual tumor deposits or pathologic lymph nodes may remain in the mesorectum after neoCRT, which undoubtedly affects the accuracy of cCR assessment, the choice of treatment strategy, and the clinical outcomes^30^. In our study, RCCs were found in a nearby lymph node in 1 of the 52 patients (1.9%), with no RCCs in the bowel wall; this statistic was slightly lower than the result reported by Duldulao MP *et al.* (4.7%)^20^. This result is also a notable challenge for an organ-sparing approach after neoCRT. Moreover, other factors may also affect the final pathological results of biopsies, such as the operator’s experience and technique, and the quantity of biopsy specimens.

Our findings have important clinical significance. The preferential distribution of RCCs in the layers of the muscularis propria and subserosa/perirectal fat implies that more attention should be paid when nonsurgical therapy is an option for any patient after neoCRT; additionally, full thickness transanal excision may be difficult in the R0 resection of the primary lesion, although some studies have shown that full thickness transanal excision is an option for patients with a major clinical response after neoCRT^31^,^32^. Biopsy of the primary rectal lesion after neoCRT may not be routinely recommended because it cannot be used to identify patients with pCR. Future studies should focus on the combination of a series of clinical examinations and the identification of predictive biological markers for neoCRT, such as mutations in the K-ras and p53 genes^33^, single-nucleotide polymorphisms of superoxide dismutase 2 and interleukin- 13^15^,^27^, and a series of blood biomarkers such as CEA and IL-8^34^.

A few limitations of this study should be addressed. First, because this was a single-center retrospective study, a potential selective bias cannot be excluded. Large prospective multicenter studies may be needed to further validate these results. Second, the lack of uniformity in the protocols and times of chemotherapy should also be mentioned, which may have effects on the distribution of RCCs in each layer of the bowel wall. Third, just as some studies have shown that the interval between neoCRT and surgery may affect tumor response^25^,^26^, different median intervals of the postneoadjuvant therapy biopsy (37 days) and the median interval of surgery (53 days) may affect the consistency of the pathological results between biopsy specimens and surgical specimens. Fourth, the 7th version of the TNM staging system was used to evaluate the extent of the residual tumor in the surgical specimens, which may be confusing when the ypN1c and ypT3 category is identified: tumor deposits in the mesorectum are diagnosed as ypN1c, whereas such deposits may represent primary tumor fragmentation after neoCRT (i.e., classified as ypT3 and not ypN1c). However, in this study, only 8 cases were categorized as ypN1c, and the stages of the primary lesions were as follows: 1 case with ypT2 disease and 7 with ypT3 disease. Therefore, the confusion of postchemoradiotherapy staging may have exerted a very limited influence on our final results because most of the patients had ypT3 disease.

## Conclusion

After preoperative VMAT with concurrent chemotherapy, the RCCs in the different layers of the bowel wall are distributed preferentially in the layers of the muscularis propria and subserosa/perirectal fat. The sensitivity of biopsy specimens for predicting the pathological response after chemoradiotherapy and prior to surgery was very low. A conventional endoscopic biopsy of a primary rectal lesion after neoCRT may not be routinely recommended because this method cannot be used to accurately identify patients who achieved a pCR.

## Additional Information

**How to cite this article**: Xiao, L. *et al.* Pathological Assessment of Rectal Cancer after Neoadjuvant Chemoradiotherapy: Distribution of Residual Cancer Cells and Accuracy of Biopsy. *Sci. Rep.* **6**, 34923; doi: 10.1038/srep34923 (2016).

## Figures and Tables

**Table 1:** Comparison of the histopathological results between the biopsy specimens and the surgical specimens of the 79 patients.

Biopsy specimen histology type	Cases (n, %)	Surgical specimen histology type (n, %)		
		No cancer cells (ypCR)	Adenocarci -noma	Carcinoma *in situ*
Definite cancer cells	8 (10.1)	0	7 (87.5)	1 (12.5)
Suspicious cancer cells	5 (6.3)	1 (20)	4 (80)	0
No cancer cells	66 (83.5)	16 (24.2)	50 (75.8)	0
Total	79	17 (21.5)	61 (77.2)	1 (1.3)

The consistency rate between the biopsy specimens and the surgical specimens was only 30.4% (24/79). Of these, 16 contained no cancer cells and 8 displayed definite cancer cells. The sensitivity of the biopsy specimens in predicting the pathological response was only 12.9% (8/62), whereas the specificity was 94.1% (16/17). Of the 62 patients with non-pCR, 54 patients (87.1%) demonstrated inconsistent pathological results between the biopsy specimen and the surgical specimen.
